# Development of Automated Triggers in Ambulatory Settings in Brazil: Protocol for a Machine Learning–Based Design Thinking Study

**DOI:** 10.2196/55466

**Published:** 2024-08-12

**Authors:** Claire Nierva Herrera, Fernanda Raphael Escobar Gimenes, João Paulo Herrera, Ricardo Cavalli

**Affiliations:** 1 Fundamental of Nursing Ribeirão Preto College of Nursing University of São Paulo Ribeirão Preto Brazil; 2 Eldorado Research Institute Campinas Brazil; 3 Faculty of Medicine of Ribeirão Preto University of São Paulo Ribeirão Preto Brazil

**Keywords:** machine learning, ambulatory care, patient safety, medical records systems, computerized, patient safety, technology, quality of care, automated triggers, limitation, predict, potential risk, outpatient, ambulatory patient, walk-in, adverse events, evidence-based, preventive, low-income countries, middle-income countries, data, scarcity, standardization, quality intervention

## Abstract

**Background:**

The use of technologies has had a significant impact on patient safety and the quality of care and has increased globally. In the literature, it has been reported that people die annually due to adverse events (AEs), and various methods exist for investigating and measuring AEs. However, some methods have a limited scope, data extraction, and the need for data standardization. In Brazil, there are few studies on the application of trigger tools, and this study is the first to create automated triggers in ambulatory care.

**Objective:**

This study aims to develop a machine learning (ML)–based automated trigger for outpatient health care settings in Brazil.

**Methods:**

A mixed methods research will be conducted within a design thinking framework and the principles will be applied in creating the automated triggers, following the stages of (1) empathize and define the problem, involving observations and inquiries to comprehend both the user and the challenge at hand; (2) ideation, where various solutions to the problem are generated; (3) prototyping, involving the construction of a minimal representation of the best solutions; (4) testing, where user feedback is obtained to refine the solution; and (5) implementation, where the refined solution is tested, changes are assessed, and scaling is considered. Furthermore, ML methods will be adopted to develop automated triggers, tailored to the local context in collaboration with an expert in the field.

**Results:**

This protocol describes a research study in its preliminary stages, prior to any data gathering and analysis. The study was approved by the members of the organizations within the institution in January 2024 and by the ethics board of the University of São Paulo and the institution where the study will take place. in May 2024. As of June 2024, stage 1 commenced with data gathering for qualitative research. A separate paper focused on explaining the method of ML will be considered after the outcomes of stages 1 and 2 in this study.

**Conclusions:**

After the development of automated triggers in the outpatient setting, it will be possible to prevent and identify potential risks of AEs more promptly, providing valuable information. This technological innovation not only promotes advances in clinical practice but also contributes to the dissemination of techniques and knowledge related to patient safety. Additionally, health care professionals can adopt evidence-based preventive measures, reducing costs associated with AEs and hospital readmissions, enhancing productivity in outpatient care, and contributing to the safety, quality, and effectiveness of care provided. Additionally, in the future, if the outcome is successful, there is the potential to apply it in all units, as planned by the institutional organization.

**International Registered Report Identifier (IRRID):**

PRR1-10.2196/55466

## Introduction

### Overview

In low- and middle-income countries, the burden of poor-quality health care has not been adequately quantified due to data scarcity, lack of standardization, and insufficient research on quality interventions. Moreover, recent estimates suggest that in these countries, between 5.7 and 8.4 million people die annually due to adverse events (AEs), where an incident resulted in harm to a patient [1]. Among the common AEs that can lead to preventable harm to patients are medication errors, unsafe surgical procedures, health care–associated infections, diagnostic errors, patient falls, pressure injury, patient misidentification, unsafe blood transfusions, and venous thromboembolism [1,2]. Annually, the indirect costs of harm from unsafe health care practices amount to trillions of US dollars worldwide, with 4 out of every 100 patients in low-to-middle-income countries losing their lives due to substandard [3]. According to the World Health Organization (WHO), the financial and economic costs of safety lapses in the health care sector will be even greater and will remain a challenge for modern health care delivery in all countries. This is attributed to patient safety incidents resulting in fatalities, disabilities, and distress for both patients and their families, along with health care workers involved in serious patient incidents [2]. This situation reinforces the hypothesis that AEs are a significant yet neglected public health issue. Furthermore, during the 72nd World Health Assembly held in May 2019, the WHO recognized patient safety as a global health priority and an essential component for achieving universal health coverage across all countries. The WHO, in collaboration with Member States, adopted resolution WHA72.6 “Global Action on Patient Safety,” with the objective of transforming the movement into a social action where patients start demanding safer health care. This marks a strategic moment as this action has been globally acknowledged as a powerful policy tool that will shape the global patient safety agenda in the coming years and reduce the costs related to AEs at all levels of health systems [1].

There are several methods available for investigating and measuring AEs, such as voluntary reporting, retrospective review of medical records involving the analysis of past patient records, prospective analysis of the patient charts of inpatients which includes capturing real-time data, direct observation, interviews with patients and health care professionals, and analysis of indicators, reports, and complaints of poor practices [4]. Among these methods, one of the most used inpatient safety research is voluntary reporting, which relies on health care providers reporting AEs that they encounter during patient care [5,6]. Another frequently used methodology in research is manual and retrospective medical record review [5,7-10]. This method is considered the “gold standard” for estimating the occurrence of AEs in hospitalized patients due to its ability to capture detailed information and this involves trained reviewers examining patient charts to identify potential AEs, followed by physician review to determine if an AE indeed occurred [11].

In the year 2003, the Institute for Healthcare Improvement (IHI) developed the Global Trigger Tool for Measuring Adverse Events [8]. This approach involves external reviewers who gather information through triggers, which are clues or indicators recorded in archived medical records that signal the potential occurrence of an AE associated with patient care [7,8]. In 2001, the IHI proposed triggers focused on tracking AEs in the outpatient setting [8], known as the Outpatient Adverse Event Trigger Tool [12]. It is important to note that the first step of the patient safety research cycle proposed by the WHO involves measuring the magnitude of AEs [1].

Despite the advantages of trigger tools in AE tracking, the methodology presents disadvantages including variation in detected AEs [6,13], limited scope [7,10], resource-intensive usage [14-16], lack of specificity [16,17], delay in tool application [7,10,15,16], dependency on complete and legible documentation [7,18], reliance on human judgment [15,16], limited data extraction [15], need for data standardization [16], and limited sensitivity [7,10,18]. For these reasons, researchers have proposed the use of automated triggers to overcome the limitations of the manual method. In investigations conducted in the United States [14] and Switzerland [16,18], researchers used automated triggers to track AEs.

In another study, machine learning (ML) was used to assess risk factors associated with severe AEs, after tracking patient harm in a cancer hospital in Chongqing, China [19]. The findings indicated that automated triggers and ML successfully identified risk factors for severe AEs induced by antineoplastic drugs in patients with cancer. The ML model incorporated variables such as age, cancer type, number of prescribed medications, and other factors. The study’s outcomes were considered promising, as the developed model demonstrated the ability to predict severe AEs induced by antineoplastic drugs in patients with cancer. The researchers concluded that the use of automated triggers and ML is a promising approach to identifying risk factors for severe events, in contrast to the manual and retrospective method which aims to track events that have already occurred. Consequently, automated triggers represent a proactive approach, while triggers developed by the IHI are reactive.

### Theoretical and Methodological Framework

#### Using Design Thinking Framework

Design thinking is defined as a human-centered approach to solving complex problems and finding innovative solutions [20]. Its objective is to creatively address adversities, focusing on the needs of the users [20]. The method has been used in the health care field with the purposes of enhancing the patient’s experience, developing solutions for clinical complications, creating business models in health, and improving service efficiency [20]. Previous research results have demonstrated that the use of design thinking in health care can lead to bold and people-centered solutions, as well as improvements in the efficiency of services provided to patients [20].

The method consists of the phases of (1) empathize and define the problem, which involves observations and questioning to understand both the user and the challenge at hand; (2) ideation, where various solutions to the problem are generated; (3) prototyping, which involves building a minimal representation of the best solutions; (4) testing, where user feedback is obtained to refine the solution; and (5) implementation, where the refined solution is tested, the change is evaluated, and scaling is considered. This phase may lead to new discoveries and the continuation of the process [20].

Recent research conducted in the health care field [21-24] with the purpose of applying design thinking in clinical research [25,26], patient safety, and quality of care [27], as well as in the education of health care professions [21-24], demonstrated that the method has contributed to the development of simple yet efficient solutions for managing problems. For example, it has been effective in reducing the outbreak of the COVID-19 pandemic [24], improving health research reporting [26], enhancing clinical researchers’ understanding of issues in dementia care [25], refining patient safety and overall quality of care in the operating room [27], proposing ethical implications for organ transplantation [21], and fine-tuning curricula and educational programs for health care professions [22].

#### ML in the Health Care Field

In recent years, there has been an increased interest in ML in the health care field, driven in part by the availability of large data sets and the development of powerful computing resources. ML, a branch of artificial intelligence (AI) and computer science, focuses on using data and algorithms to mimic the way humans learn, gradually improving its accuracy. The method is being applied to a wide range of fields, including health and finance [28,29]. With the rise of AI and the growing influx of digital data in health care, new tools have emerged that can enhance patient care and alleviate the labor-intensive nature of nursing work [28,29]. It is anticipated that in the next decade, health care delivery will be revolutionized by the integration of digital health technologies and AI [29].

Recent research using ML in the health care field, such as the development of an early warning system for sepsis [30], the assessment of mortality risk in older adults [31], the prediction of hospital admissions in emergency departments [32], and the forecast of AEs after percutaneous coronary intervention [33], has concluded that these techniques are effective in predicting such events and can provide increased accuracy in outcomes.

#### Goals and Research Question

This protocol describes a methodology designed using design thinking frameworks to develop ML-based automated triggers for outpatient health care settings in Brazil and overcome the limitations of manual methods. Additionally, the specific objectives are (1) to apply the principles of design thinking in the creation of automated triggers; (2) to gain insights into risk factors for AEs and limitations in early detection of health care–related harm from the perspectives of health care professionals, patients, and family or caregivers; (3) to create automated triggers through ML; and (4) to evaluate the implementation of this solution in the real world. Considering the foregoing, the question arises if it is possible to develop automated triggers through ML to predict early risks of avoidable harm related to outpatient health care.

## Methods

### Study Design and Setting

Mixed methods research will be conducted within a design thinking framework. The study will take place at the outpatient unit of a prominent tertiary-level teaching hospital in the Metropolitan Region of Ribeirão Preto, located in the state of São Paulo, Brazil. The institution provides services across various medical specialties, offering exclusive services to the Unified Health System. Within the outpatient building is the institution’s largest surgical center, as well as the Central Laboratory Division—the first public laboratory in Brazil certified by the American College of Pathologists. The unit also has jointly a pharmaceutics unit, responsible for dispensing prescribed medications and regularly producing various categories of pharmaceuticals.

### Participants

#### Study Inclusion and Exclusion Criteria

In this study, exclusively included will be adult patients aged 18 years or older, fluent in Brazilian Portuguese, along with their family members or caregivers and members of the multiprofessional health care team (physicians, nurses, nursing technicians or assistants, pharmacists, nutritionists, physiotherapists, psychologists, and speech therapists) with a work experience in the outpatient unit exceeding 1 year. Residents and health care professionals on vacation or leave during the data collection period will be excluded. Regarding patients’ electronic medical health records (EMHRs), those originating from pregnant individuals and psychiatric patients will be excluded. Additionally, patients’ EMHRs with insufficient and incomplete information will be excluded.

#### Sample Size

The sample size will be divided into 2 stages. For the interview, a sample size of 20 participants will be considered, including patients, family members or caregivers, and members of the multidisciplinary health care team, taking into account the concept of data saturation [34,35]. Finally, for predictive analysis in ML for patients’ EMHRs, the sample size will depend on the number of variables identified and included for triggers that need to be considered [36].

### Ethical Considerations

The study will be conducted in accordance with the Declaration of Helsinki and in compliance with the standards and guidelines established by the National Research Ethics Commission, as specified in the regulations and recommendations of Resolution 466/12 of the National Health Council [37], which regulates research involving human participants.

This project also involves documentary research as the incident or AE reports that occurred with ambulatory patients will be analyzed. It will not be possible to obtain informed consent from individuals whose data are contained in these restricted-access documents, as they do not attend the owning institution. In this case, the researcher will sign the Commitment Term for Data Use and Handling, which will be handled only after approval by the Research Ethics Committee and the owning institution.

The study was approved by the members of the organization within the institutions (146.00010241/2023-15) and it has been approved by the College of Nursing, Ribeirão Preto, University of São Paulo (Brazilian Ethics Board 6.811.085) and by the ethics board of the institution where the study will take place (Brazilian ethics board 6.851.281), enabling the commencement of data gathering in the ambulatory care unit.

Participants will be informed about the study’s objectives and, upon agreeing to participate voluntarily, they will be asked to sign the informed consent form. There will be 3 informed consent processes—2 separate informed consent for stages 1 and 2 (1 for patients, family members or caregivers, and another for participants who are members of the multidisciplinary health care team). The third informed consent will be considered for stage 4 participants in this study.

Participants will not receive direct benefits or compensatory benefits, but the results of this study may generate indirect benefits. The results will be useful for developing automated triggers in the outpatient setting. As mentioned in stage 1, under step 4, participants’ identification will be encrypted, to guarantee privacy and will not be disclosed when publishing the study results. All data obtained will be used for scientific purposes only.

### Procedures for Data Collection and Data Analysis

#### Overview

**Figure 1:**
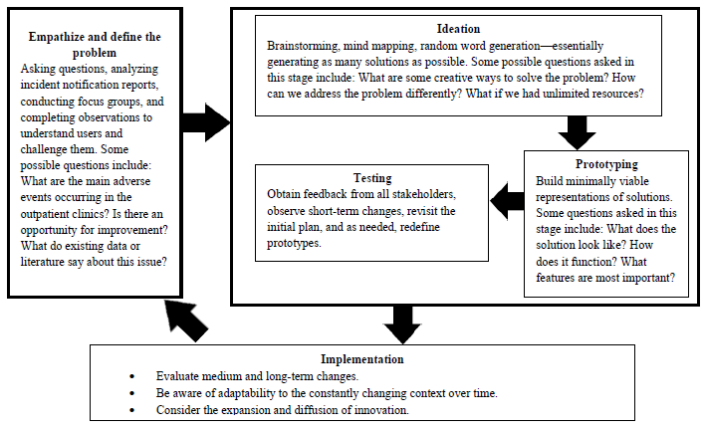
Stages of design thinking (adapted from Altman M et al [20], which is published under Creative Commons Attribution 4.0 International License [38].

This phase will be divided into 5 stages based on design thinking, as presented in Figure 1 [20].

#### Empathize and Define the Problem: Stage 1

##### Overview

In this stage, qualitative research will be conducted. The researcher will meet with stakeholders to collect data on their perceptions of a specific problem and conduct on-site observations to understand users and challenges at hand. In addition, the institution’s incident notification reports will be analyzed to map the most frequent types, severity according to the WHO classification [39], and prevalence.

To access incident or AE reports from the institution and conduct interviews, the researcher will contact the unit manager and the institution’s risk management representative to present the research project and clarify its objectives. After obtaining proper authorization from the unit manager and the institution’s risk management, the risk management manager will proceed to provide tabulated data, which includes the variable of interest. These variables include the patient’s registration number, patient characterization information, type of event, severity or degree of resulting damage or harm, consequences of the damage or harm, and the occurrence location, specifically within which medical specialty. Also, during these visits, with the purpose of analyzing AE notification reports and mapping the patient’s journey from admission to discharge. Furthermore, “extreme patients” will be identified, those with multiple admissions in a short period. Additionally, the EMHR will be examined to characterize patients who have experienced AEs, defined as incidents resulting in patient harm due to health care [1], such as medication errors and readmissions within 30 days.

Subsequently, authorization will be requested to access patients, family members or caregivers, and health care professionals working in the unit, who will be invited to participate voluntarily and in writing. The researcher will schedule interviews within the unit, in a reserved location agreed upon with the manager, so as not to interfere with the institution’s care routine. In this case, semistructured interviews will also be conducted, recorded in audio and later transcribed [40] to understand the experiences of patients, family members or caregivers, and health care professionals regarding AEs related to care provided in the outpatient setting.

An interview protocol with 4 to 5 questions will be considered [40]. Then, participants will be asked to elaborate on their ideas or what they said in detail [40]. The interviews will be fully transcribed and later analyzed with the assistance of the ATLAS.ti software (ATLAS.ti Scientific Software Development GmbH) [41]. Data will be analyzed following the process [40] that involves the following steps.

##### Step 1

Organize and prepare data for analysis—involves transcribing interviews or classifying and organizing data into different types, depending on the sources of information.

##### Step 2

Read all data—the first step is to get a general idea of the information and reflect or begin to record general thoughts about the data at this stage.

##### Step 3

Start detailed analysis with a coding process—involves taking collected text data, segmenting sentences (or paragraphs) into categories, and labeling these categories with a term, often a term based on the participant’s actual language.

##### Step 4

Use the coding process, as well as categories or themes for analysis—involves a detailed representation of information about people, places, or events in an environment. Then use coding to generate a small number of themes or categories, which should show multiple participant perspectives and be supported by diverse quotations and specific evidence.

##### Step 5

Move forward in how the description and themes will be represented—the most popular approach is to use a narrative text to convey the results of the analysis, which can be a discussion mentioning a timeline of events, a detailed discussion of various themes (with subthemes, specific illustrations, multiple perspectives from individuals, and quotes), or a discussion with interconnected themes. Visuals, figures, or tables are also used as supplements to discussions.

##### Step 6

Make an interpretation or meaning of the data—capturing the essence of the idea and lessons can be the researcher’s personal interpretation, inserted into the understanding that the researcher brings to the study from their own culture, history, and experiences. It can also be a meaning derived from comparing results with information obtained from literature or theories. Thus, the results confirm past information or deviate from it. It can also suggest new questions that need to be asked—questions raised by the data and analyses that the researcher had not previously anticipated in the study. Based on the themes identified from the qualitative data analysis, the researcher will identify the most common AEs in outpatient care and their likely causes. In this stage, the Consolidated Criteria for Reporting Qualitative Research (COREQ) guideline [42] will be adopted.

#### Ideation: Stage 2

In this phase, the probable causes of the most common and severe AEs in outpatient care will be explored from the perspective of health care professionals and users or patients, and family members or caregivers. To achieve this, the methodology of root cause analysis will be used, using an Ishikawa diagram, also known as a cause and effect or fishbone diagram. This tool enables the systematic categorization and identification of the root causes of a problem. Through this diagram, it becomes possible to visualize and analyze the various variables that may be contributing to the issue at hand, facilitating the identification of its primary causes [43]. It is noteworthy that the patient’s journey in the service will also be mapped, and subsequently, a process flowchart will be developed.

Based on the results from stages 1 and 2, as many solutions as possible will be generated to address the challenge of developing automated triggers capable of predicting potential risks of harm to ambulatory patients.

#### Prototyping: Stage 3

**Figure 2:**
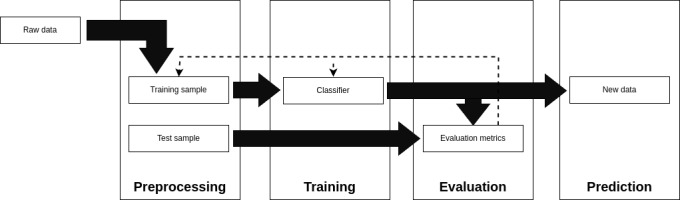
Application scheme of machine learning algorithms.

In this phase, a patient-centered approach will be applied to create automated triggers capable of identifying risks of avoidable harm. To achieve this, ML techniques will be adopted as illustrated in Figure 2. Moreover, a separate paper focused on explaining the method of ML in detail will be applied after the outcomes of stages 1 and 2, as well as the access gained to patients’ EMHRs in this research project.

The ML models are mathematical models whose parameters are adjusted based on the examples presented during the training phase [28]. Before training, the data must undergo preprocessing to standardize and eliminate any inconsistencies. Removing duplicate entries, correcting missing values, converting categorical data into numbers, and filtering relevant attributes are examples of data preprocessing techniques. At this stage, the subsets of samples to be used for model training and evaluation must also be defined. To avoid bias in learning, samples will be randomly selected.

From the preprocessed data, a classification model will be trained to indicate the risk of an AE (output variable) based on specific triggers (input variables). The model selection will be based on the data distribution, which should be interpreted by an expert in the field. Gradient boosting–based models [44-46] and logistic regression [44,45,47] are examples of modern ML algorithms well-suited for classification tasks involving tabular data. Finally, the trained model will be used to identify or “predict” the most likely outcomes of new data not present in the training data set [28].

Quantitative evaluation metrics such as calibration, discrimination, sensitivity, and specificity will be used to quantitatively assess the model’s performance from the evaluation subset [36]. It is emphasized that the entire process involving ML techniques will involve collaboration with an expert in the field of AI. Additionally, the Transparent Reporting of a Multivariable Prediction Model for Individual Prognosis or Diagnosis (TRIPOD) guideline [48] will be followed with the aim of reporting an AI-related intervention, also considering the Guidelines for Developing and Reporting Machine Learning Predictive Models [49].

#### Testing: Stage 4

In this stage, the prototype is tested with health care professionals as participants to obtain feedback and identify areas for improvement. For this purpose, the think aloud method will be used [50]. Initially, participants will receive a brief explanation of the technique and the tasks they should perform. While participants perform the tasks, they must verbally report everything they are thinking and doing, from the moment they start the task until they complete it.

The researcher will take careful notes on the observations made by participants in a field diary. After completing the task using the think aloud technique, the researcher will analyze the observations and identify patterns of behavior and common problems encountered by participants during the interaction with the user interface. Finally, the researcher will prepare a report describing the observations made, including the identified problems and possible solutions to these problems.

#### Implementation: Stage 5

**Figure 3:**
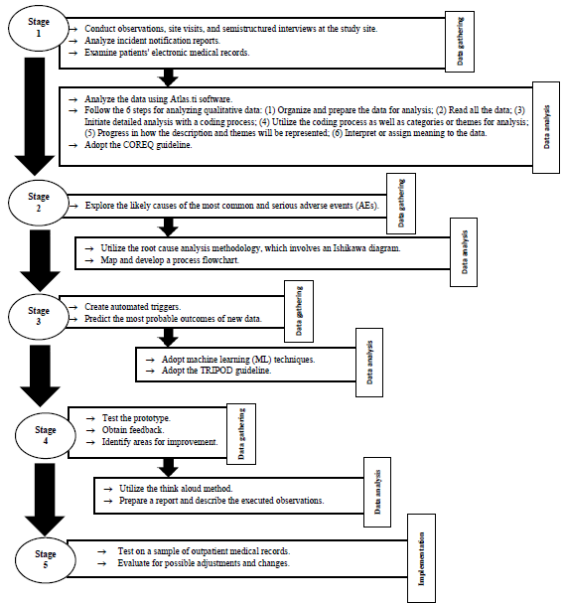
Operationalization of the study, including data collection and data analysis. COREQ: Consolidated Criteria for Reporting Qualitative Research; TRIPOD: Transparent Reporting of a Multivariable Prediction Model for Individual Prognosis or Diagnosis.

In this final phase, the algorithms developed through the ML technique, in collaboration with an expert in the field of ML, will be tested on a sample of patient records treated in outpatient care and evaluated for possible adjustments and changes. Additionally, this stage may result in new discoveries and the continuation of the process. In Figure 3, the operationalization of the study is illustrated, including data collection and data analysis.

## Results

This protocol describes a research study in its preliminary stages, prior to any data gathering and analysis. The research is currently in its initial phases, with the institution’s organization showing support for the project. As mentioned previously, the study was approved by the members of the organization within the institutions in January 2024 and in May 2024, it was approved by the ethics board of the University of São Paulo and the institution where the study will take place.

After approval by the ethics board, stage 1, commenced with data gathering for qualitative research in June 2024. The researcher conducted a brief meeting with the unit manager and the risk management manager. Site observation began and as of June 17, 2024, semistructured interviews were initiated and ended on June 28, 2024, with 25 participants having participated in individual interview, taking into account the concept of data saturation. Data analysis has begun, considering the first step, which involves organizing and preparing data for analysis, including transcribing interviews. If the data information is not enough, follow-up questions will be added for the individual participants. Moreover, tabulated data of notification reports will be collected from August 12 to 31.

Additionally, funding for this study was granted by the National Council for Scientific and Technological Development (Conselho Nacional de Desenvolvimento Científico e Tecnológico) under process 141042/2024-9 as of May 2024. The study was granted funding in the Doctoral Sandwich Program Abroad (Programa de Doutorado Sanduíche no Exterior), process (88881.982930/2024-01), which aims to support research projects in higher education and research institutions outside Brazil, starting from November 2024 until April 2025 in Finland.

The abstract of this study was selected for presentation during the 35th International Nursing Research Congress from July 2024 to August 2024 in Singapore, organized by Sigma Global Nursing Excellence.

As this is the early stage of the research project and stage 3, which involves prototyping based on the framework of design thinking, will entail the creation of automated triggers using the method of ML, it can only present some possible models that will be used. The possible models will depend on the outcomes of stages 1 and 2 of design thinking, as well as the access gained to patients’ EMHRs. Moreover, it will depend on the number of variables identified and included for triggers that need to be considered. A separate paper focused on explaining the method of ML can be considered after the outcomes of stages 1 and 2 in this research project.

## Discussion

### Principal Findings

In this paper, we present the research protocol in its preliminary stages, prior to any data gathering and analysis. This study is the first to create automated trigger tools in ambulatory settings in Brazil, presenting a valuable contribution to the health field while also adapting the design thinking framework. This framework is still underexplored in Brazilian research, especially in the context of patient safety. It is crucial to highlight the real-world application, specifically the effective implementation in the local realities.

### Comparison to Prior Work

In Brazil, despite a few published studies on the application of the trigger tool, most have focused on hospital settings [51,52], and more recently, on dental care [53]. However, according to the 2018 report from the Organization for Economic Cooperation and Development [54], about half of the global burden of AEs originated in primary health care, and 4 in 10 patients experience safety incidents, often resulting in hospitalizations and increased care needs. Moreover, the most common AEs in primary health care are related to medication use, largely caused by polypharmacy.

Given this context, in 2001, IHI proposed triggers for tracking AEs in the outpatient setting [12], known as the Outpatient Adverse Event Trigger Tool [55]. The tool comprises 11 triggers that provide clues or hints regarding the existence of an AEs in the patient’s medical record, including (1) new cancer diagnosis, (2) placement in a nursing home, (3) hospital admission and discharge, (4) 2 or more outpatient visits in a year, (5) surgical procedure, (6) emergency department visit, (7) use of more than 5 medications, (8) change of physician, (9) complaint letter, (10) more than 3 nursing calls in a week, and (11) abnormal laboratory value. These triggers were tested in American outpatient clinics but are certainly not the only possible triggers, although they represent an important starting point. According to IHI [8], each institution should choose to add, remove, or modify the triggers currently on the list to meet local realities. However, this study will develop automated triggers, in contrast to the manual and retrospective methods.

### Strengths and Limitations

This study has the potential to positively impact the quality of care provided and simultaneously make a valuable contribution to the international scientific community. Thus, the best practices for outpatient care encompass principles of care continuity beyond hospital walls. In line with these observations, the importance and urgency of identifying events related to unsafe care in the outpatient context are emphasized to guide improvement and safety strategies for patients in these health care settings [56]. Furthermore, decisions can be made to minimize the burden of preventable harm, both economically and socially [57], as well as to promote a safety culture and establish high-reliability organizations.

This study will be conducted in an ambulatory setting with participants who are adults aged 18 years or older. It will exclude EMHR from pregnant individuals and psychiatric patients, which are anticipated limitations of this study.

### Future Directions

For this study protocol, a separate paper focused on explaining the ML methods can be considered after the outcomes of stages 1 and 2 in this study. In the future, it will be used in every unit and will adjust the triggers based on each unit of the hospital. As mentioned, this is the first study to create automated triggers in Brazil. Therefore, there is a potential high demand for contributions nationally, and our study will also benefit other lines of research both nationally and internationally.

### Conclusions

After the development of automated triggers in the outpatient setting, it will be possible to prevent and identify potential risks of AEs more promptly, providing valuable information. This technological innovation not only promotes advances in clinical practice but also contributes to the dissemination of techniques and knowledge related to patient safety. Additionally, health care professionals can adopt evidence-based preventive measures, reducing costs associated with AEs and hospital readmissions, enhancing productivity in outpatient care, and contributing to the safety, quality, and effectiveness of care provided. Additionally, in the future, if the outcome is successful, there is the potential to apply it in all units, as planned by the institutional organization.

## Abbreviations

AE: adverse event
AI: artificial intelligence
COREQ: Consolidated Criteria for Reporting Qualitative Research
EMHR: electronic medical health record
IHI: Institute for Healthcare Improvement
ML: machine learning
TRIPOD: Transparent Reporting of a Multivariable Prediction Model for Individual Prognosis or Diagnosis
WHO: World Health Organization

## Data Availability

The data sets generated and analyzed during this study are available from the corresponding author on reasonable request.
